# Comparative Analysis of Hip Abductor Strength Following Anterolateral Versus Posterior Approaches in Total Hip Replacement: A Prospective Study

**DOI:** 10.7759/cureus.96757

**Published:** 2025-11-13

**Authors:** Madhuresh Kumar, Digvijay Agarwal, Amit Sharma

**Affiliations:** 1 Department of Orthopaedics, Himalayan Institute of Medical Sciences, Dehradun, IND; 2 Department of Physiotherapy, Himalayan Institute of Medical Sciences, Dehradun, IND

**Keywords:** anterolateral approach, hip abductors, muscle strength recovery, posterior approach, total hip arthroplasty

## Abstract

Background

Total hip replacement (THR) is a highly successful procedure for end-stage hip disorders; however, persistent postoperative functional limitations continue to be reported, particularly due to weakness of the hip abductor musculature, which plays a critical role in pelvic stability and gait mechanics. Surgical approach may influence abductor integrity and postoperative recovery. This study prospectively evaluated hip abductor muscle strength recovery following THR and compared outcomes between the anterolateral and posterior approaches.

Methodology

A prospective, observational study was conducted among 50 THR patients at a tertiary care center over 12 months. Patients were allocated to anterolateral (n = 25) or posterior (n = 25) approaches based on surgeon preference. Baseline demographic, clinical, and radiological profiles were comparable between groups. Hip abductor strength was assessed using Manual Muscle Testing (MMT) at 48 hours, 6 weeks, and 3 months postoperatively. A standardized rehabilitation protocol was implemented for all patients starting 48 hours after surgery. Data were analyzed using SPSS version 27.0 (IBM Corp., Armonk, NY, USA), and a p-value <0.05 was considered statistically significant.

Results

Abductor strength improved significantly from 48 hours to 3 months in the entire cohort (p = 0.001). At all follow-up intervals, the posterior approach demonstrated significantly higher mean abductor strength than the anterolateral approach (48 hours: 2.60 ± 0.70 vs. 2.04 ± 0.35; 6 weeks: 3.52 ± 0.58 vs. 2.72 ± 0.54; 3 months: 4.48 ± 0.58 vs. 3.52 ± 0.51; p = 0.001 for all). Baseline clinical examination parameters showed no statistically significant differences between groups, suggesting comparability at entry. These findings indicate that early postoperative functional abductor recovery is more favorable with the posterior approach.

Conclusions

The posterior surgical approach was associated with superior early hip abductor muscle strength recovery when compared to the anterolateral approach following THR. While these results have important implications for functional rehabilitation and approach selection, interpretation should be made cautiously due to non-randomized allocation and modest sample size. Future studies with larger cohorts, longer follow-up, and objective quantitative strength assessment (e.g., dynamometer measurement) are warranted to validate these findings and guide evidence-informed approach selection in THR.

## Introduction

Total hip replacement (THR) is a widely performed and highly effective surgical intervention for end-stage hip osteoarthritis and avascular necrosis (AVN) of the femoral head, offering pain relief, improved function, and enhanced quality of life [1]. In India, most patients undergoing THR present with hip arthritis due to femoral neck fractures, AVN, or seronegative arthritis [2]. Globally, the number of THR procedures is increasing rapidly, with over 500,000 expected annually in the coming years [3].

The rise in THR can be attributed to greater life expectancy, better public awareness, improved surgical techniques, and wider access to healthcare through initiatives such as Ayushman Bharat (a government-funded national insurance scheme that improves access to tertiary care surgical interventions in India). Lifestyle factors such as osteoporosis, obesity, alcohol use, and trauma further contribute to the increasing demand [4].

Surgical success depends on factors such as patient selection, prosthesis choice, and precise surgical technique. A watertight anatomical closure is crucial in minimizing complications such as dislocation and hematoma by preserving joint stability and reducing dead space [5]. Despite high satisfaction rates, with over 90% of patients reporting favorable outcomes, functional limitations persist, especially in tasks such as walking and stair climbing [3]. Studies have shown a decline in stair performance five years post-THR [6] and persistent gait abnormalities even 10 years after surgery [7]. These impairments are often associated with balance deficits, postural instability, and increased fall risk [3].

Biomechanical changes post-THR, such as reduced abduction, rotation, and extension moments, are evident during walking and stair climbing. Altered gait speed and loading patterns reflect compensation strategies that affect long-term function [3]. These compensations, such as Trendelenburg gait, are associated with poor outcomes and increased mortality in older adults [3]. Functional challenges are particularly evident in activities such as stair climbing and sit-to-stand transitions, which rely heavily on hip and pelvic stability [8].

A key factor in these biomechanical changes is hip abductor muscle (HAM) dysfunction. The primary abductors, i.e., gluteus medius, gluteus minimus, and tensor fascia lata, and secondary muscles, i.e., the piriformis and sartorius [9], play a crucial role in stabilizing the pelvis during ambulation. Weakness in these muscles leads to reduced internal hip moments, decreased mobility, and lower scores on physical function scales such as the 36-Item Short Form Survey [10].

HAM weakness post-THR is also linked to limping, the Trendelenburg sign, chronic pain, and increased risk of dislocation. Treatment options vary based on injury severity, ranging from direct repair to soft tissue reconstruction or tendon transfers. Even mild abductor weakness is associated with long-term issues such as low back pain, patellofemoral pain, and knee osteoarthritis, underscoring its importance in post-THR recovery. This study aims to evaluate HAM function in THR patients during the postoperative period, comparing outcomes across surgical approaches (anterolateral vs. posterior) and rehabilitation protocols.

## Materials and methods

Study design and setting

This observational cohort study was conducted in the Department of Orthopaedics at Himalayan Institute of Medical Science, Dehradun, over a 12-month period. Institutional Ethics Committee approval was obtained (approval number: SHRU/HIMS/ETHICS/2025/394), and written informed consent was taken from all participants. In total, 50 patients scheduled for THR were enrolled consecutively. Inclusion criteria included patients with primary osteoarthritis, secondary osteoarthritis due to AVN, and femoral neck fractures. Patients with neuromuscular disorders, revision THR, or prior hip surgeries were excluded.

Sample size calculation

Based on a 95% confidence level, an expected standard deviation of 5.9 [11], and an allowable error of 1.7, the minimum calculated sample size was 46. Accounting for non-response, a total of 50 patients were included. Among them, 25 underwent THR via the anterolateral approach, and 25 via the posterior approach.

Data collection tools

Data were collected using a structured case recording form that documented patient demographics, clinical findings, diagnosis, surgical approach, and follow-up assessments. Radiographic evaluation included X-rays of the pelvis with both hips, and anteroposterior and lateral views of the affected limb to assess joint condition and postoperative implant positioning. Hip abductor strength was assessed using the Manual Muscle Testing (MMT) chart, which provided a standardized grading of muscle power during each follow-up visit.

Surgical approach and evaluation

The choice of surgical approach was based on the surgeon’s preference. Hip abductor strength was assessed using MMT. Muscle grading was done per standard criteria [12]. Postoperative hip abductor strength was assessed at three time points, namely, 48 hours after surgery, at 6 weeks, and again at 3 months. These intervals were chosen to observe both early and intermediate recovery trends in muscle function following THR. Rehabilitation began 48 hours after surgery and was performed twice daily for six weeks. The program consisted of isometric exercises for hip abduction, side leg lifts, high sitting, stationary squatting, toe-touching, and ambulation with the help of a walker.

Data analysis

Data were analyzed using SPSS version 27.0 (IBM Corp., Armonk, NY, USA). Descriptive statistics (mean, percentage, rate) were used for general data representation. Comparisons between two groups were done using unpaired t-tests, while one-way analysis of variance was used for comparisons across three time points. The chi-square test was used to compare the qualitative data. A p-value <0.05 was considered statistically significant.

## Results

The study included 50 patients with a mean age of 48.84 ± 13.89 years. Most participants (56%) were between 41 and 60 years of age, followed by 26% aged 20-40 years and 18% aged 61-80 years. Males constituted the majority (72%) of the cohort. AVN was the predominant diagnosis (78%), with smaller proportions having a neck of femur fracture (16%), secondary osteoarthritis (4%), and acetabulum fracture (2%). Comorbidities were relatively uncommon, with hypertension in 6%, and chronic kidney disease, diabetes mellitus, and rheumatoid arthritis each present in 2% of cases. There was no statistically significant difference in the demographic and clinical profile of the study population between the anterolateral and posterior approach groups (Table 1).

**Table 1:** Demographic and clinical profile of the study population.

Variable	Domain	Anterolateral approach	Posterior approach	Total	P-value
Age group	Mean age (years)	50 ± 14.38	47.68 ± 13.57	48.84 ± 13.89	0.560
	20–40 years	7 (14%)	6 (12%)	13 (26%)	0.848
	41–60 years	13 (26%)	15 (30%)	28 (56%)	
	61–80 years	5 (10%)	4 (8%)	9 (18%)	
Gender	Male	16 (32%)	20 (40%)	36 (72%)	0.208
	Female	9 (18%)	5 (10%)	14 (28%)	
Diagnosis	Avascular necrosis	17 (34%)	22 (44%)	39 (78%)	0.302
	Neck of femur fracture	6 (12%)	2 (4%)	8 (16%)	
	Secondary osteoarthritis	1 (2%)	1 (2%)	2 (4%)	
	Acetabulum fracture	1 (2%)	0	1 (2%)	
Comorbidities	Hypertension	1 (2%)	2 (4%)	3 (6%)	0.753
	Chronic kidney disease	0	1 (2%)	1 (2%)	
	Diabetes mellitus	0	1 (2%)	1 (2%)	
	Rheumatoid arthritis	0	1 (2%)	1 (2%)	

On examination, fixed flexion deformity (FFD) of the left hip was observed in three (6%) patients, with two cases showing a 20-degree deformity and one case with a 30-degree deformity. No cases of limb lengthening were noted. Limb shortening was present in 46% of patients: 22% had left-sided and 24% had right-sided shortening, with a mean shortening of 1.86 ± 0.62 cm. Neurovascular status was intact in all patients (100%). There was no statistically significant difference in the baseline clinical examination findings between the anterolateral and posterior approach groups. Although there was a quantitative difference in mean limb shortening values, this difference was not statistically significant when analyzed between groups (Table 2).

**Table 2:** Clinical examination findings.

Examination detail	Domain	Anterolateral approach	Posterior approach	Total	P-value
Deformities	FFD (20 degrees) left hip	1 (2%)	1 (2%)	2 (4%)	0.386
	FFD left (30 degrees)	1 (2%)	0	1 (2%)	
Lengthening		0	0	0	NA
Shortening	Left lower limb	7 (14%)	4 (8%)	11 (22%)	0.509
	Right lower limb	6 (12%)	6 (12%)	12 (24%)	
	Mean value (cm)	2.06 ± 0.56	1.55 ± 0.59	1.86 ± 0.62	0.039
Neurovascular status	Intact	25 (50%)	25 (50%)	50 (100%)	NA

**Figure 1:**
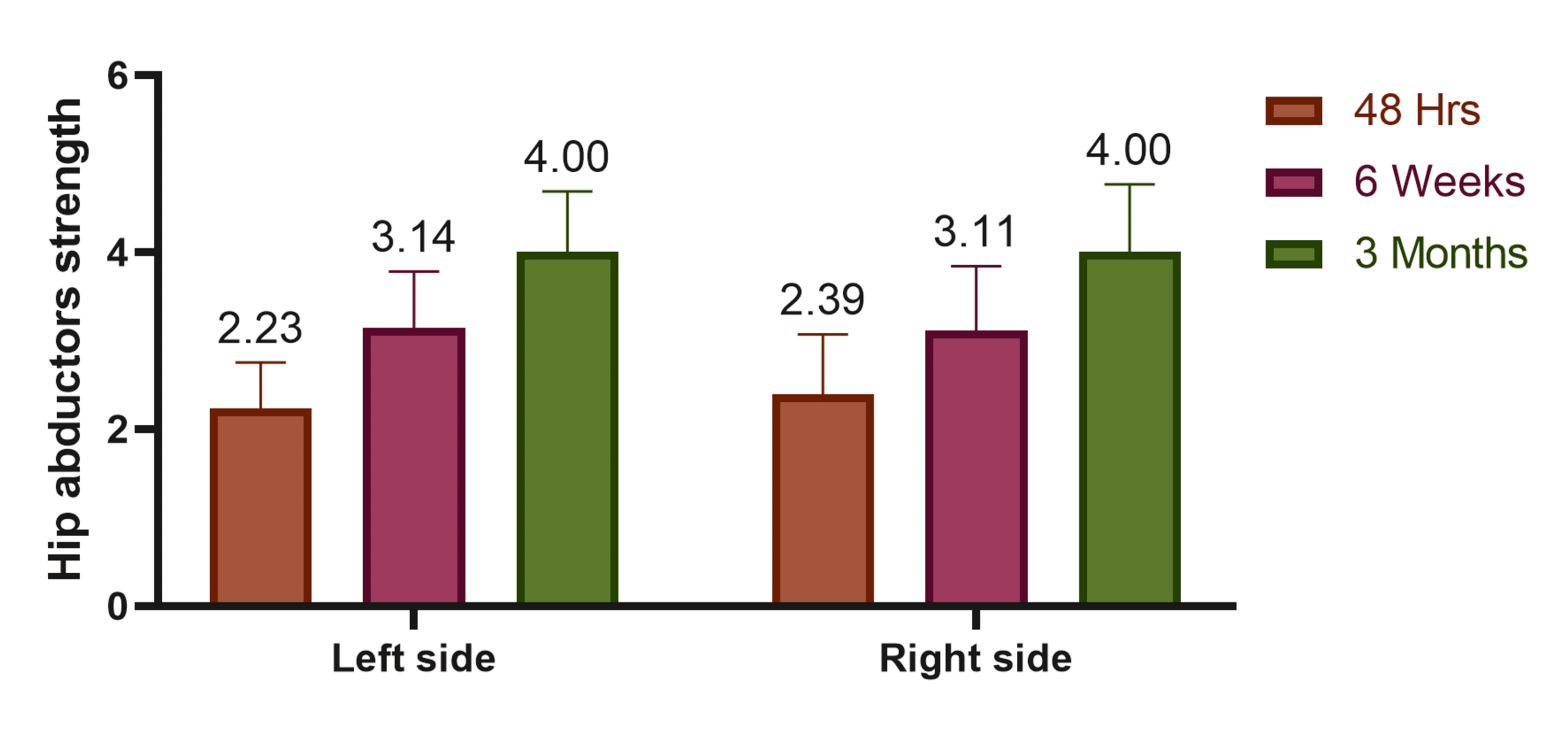
Assessment of hip abductors at follow-up visits.

Hip abductor strength improved progressively on both sides over time. At 48 hours postoperatively, the mean strength was 2.23 ± 0.52 on the left and 2.39 ± 0.68 on the right. By six weeks, it increased to 3.14 ± 0.64 (left) and 3.11 ± 0.73 (right). At three months, both sides reached a mean of 4.00. The improvement was statistically significant (p = 0.001), indicating effective postoperative recovery (Table 3, Figure 1).

**Table 3:** Hip abductor strength at follow-up visits. *: One-way analysis of variance was used to compare three means, and the unpaired t-test was used to compare two means (p < 0.05 as statistically significant).

Side	48 hours	6 weeks	3 months	F-value	P-value
Left side (n = 22)	2.23 ± 0.52	3.14 ± 0.64	4.00 ± 0.69	58.21	0.001*
Right side (n = 28)	2.39 ± 0.68	3.11 ± 0.73	4.00 ± 0.77	47.35	0.001*
t-value (left vs. right)	0.86	0.14	0.00	—	—

**Figure 2:**
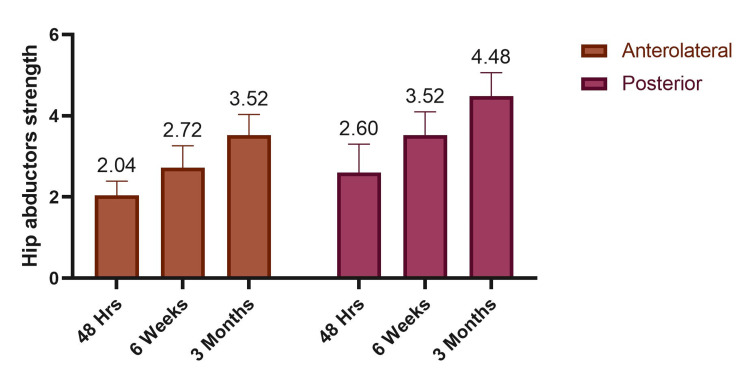
Postoperative hip abductor strength by surgical approach.

Patients who underwent the posterior approach showed significantly higher hip abductor strength at all postoperative time points compared to those with the anterolateral approach. At 48 hours, the posterior group had a mean strength of 2.60 ± 0.70 versus 2.04 ± 0.35 in the anterolateral group. This trend continued at six weeks (3.52 ± 0.58 vs. 2.72 ± 0.54) and at three months (4.48 ± 0.58 vs. 3.52 ± 0.51). The differences were statistically significant (p = 0.001), suggesting better early recovery of abductor strength with the posterior approach (Table 4, Figure 2).

**Table 4:** Postoperative hip abductor strength by surgical approach. *: One-way analysis of variance was used to compare the three means, and the unpaired t-test was used to compare two means (p < 0.05 as statistically significant).

Surgical approach	48 hours	6 weeks	3 months	F-value	P-value
Anterolateral	2.04 ± 0.35	2.72 ± 0.54	3.52 ± 0.51	76.42	0.001*
Posterior	2.60 ± 0.70	3.52 ± 0.58	4.48 ± 0.58	89.65	0.001*
P-value	0.001*	0.001*	0.001*	—	—
t-value (between groups)	3.21	4.74	6.54	—	—

## Discussion

Hip abductors are critical for maintaining hip stability and mobility. Their insufficiency, often due to degeneration or trauma during THR, can lead to gait abnormalities such as Trendelenburg gait, pain, and restricted motion [13]. Among the commonly used THR techniques, the posterior and anterolateral (Hardinge) approaches were compared in this study due to their frequent use and adequate exposure during surgery. Although literature presents mixed opinions, no approach has been definitively proven superior [14].

In our cohort, the mean age was 48.84 ± 13.89 years, with 56% of patients aged 41-60 years. Other studies have reported higher mean ages: Vaz et al. reported 64 ± 7 years [14], Downing et al. 65-67 years [15], and Whiteside and Roy 71 ± 22 years [16], while Wongsak et al. found a mean of 55.7 ± 15.1 years [13]. This suggests our population was relatively younger, possibly reflecting earlier intervention in AVN.

Males comprised 72% of our patients, which aligns with Vaz et al. (23 males, 20 females) [14]. In contrast, Downing et al. reported near-equal ratios in both surgical groups [15], and Wongsak et al. observed a predominantly female cohort (75%) [13].

The most common diagnosis in our study was AVN (78%), followed by a neck of femur fracture (16%) and secondary osteoarthritis (4%). Whiteside and Roy reported 97.49% osteoarthritis, with only 1.73% AVN cases [16], while Wongsak et al. observed 35% AVN and 60% primary osteoarthritis [13]. This highlights differing regional or institutional case mixes.

Comorbidities in our cohort were limited: hypertension (6%), chronic kidney disease, diabetes, and rheumatoid arthritis (each 2%). Downing et al. noted arthritis in 13.72-16.32% of patients [15], but specific comorbidity patterns varied.

Preoperative clinical assessments showed FFDs in 6% of patients. No cases of lengthening were noted, while 22-24% had limb shortening, with a mean of 1.86 ± 0.62 cm. Neurovascular status was intact in all. In contrast, Whiteside and Roy reported minimal limb length discrepancies (<1 cm) and a mean offset difference of 3.3 mm between operated and contralateral hips [16].

Abductor strength showed statistically significant improvement over time on both sides. On the left, it improved from 2.23 ± 0.52 at 48 hours to 4.00 ± 0.69 at 3 months. The right side showed similar gains (2.39 ± 0.68 to 4.00 ± 0.77). These results echo findings by Vaz et al., who noted functional and strength improvements at 12 and 24 weeks [14]. Downing et al. also observed increasing abductor strength up to 12 months post-THR using both approaches [15].

Wongsak et al. reported transient postoperative declines in abductor strength with the anterolateral approach, followed by significant improvements by three months [13]. Similarly, Whiteside and Roy found preserved abductor function but noted increased lateral hip pain at long-term follow-up [16].

When comparing surgical techniques, the posterior approach consistently showed superior abductor strength at all intervals. At 48 hours, strength was 2.60 ± 0.70 vs. 2.04 ± 0.35 for the anterolateral approach (p = 0.001). This trend continued at six weeks (3.52 ± 0.58 vs. 2.72 ± 0.54) and three months (4.48 ± 0.58 vs. 3.52 ± 0.51) (p = 0.001 for all). These findings align with Downing et al., who found greater strength recovery in the posterior group at 12 months [15], although the difference was not statistically significant.

Previous literature supports these observations. Mulliken et al. reported a 10% limp rate at two years with the lateral approach in 770 THRs, without comparing with the posterior approach [17]. Obrant et al. found weaker abductors with direct lateral techniques [18]. Baker and Bitounis attributed postoperative Trendelenburg signs more often to the lateral approach due to gluteal detachment [19]. Injury to the superior gluteal nerve, particularly when violating the “safe zone” within 5 cm of the greater trochanter, may also contribute to postoperative weakness [20,21]. However, Kenny et al. found that electromyography evidence of nerve injury did not always correlate with clinical weakness [22], indicating other contributing factors.

The present study has certain limitations that need to be discussed. The first limitation is that this was a non-randomized study design, which can introduce significant selection bias. Previously, Downing et al. also adopted the same study design and highlighted the risk of introducing potential selection bias and systematic differences between groups [15]. Another limitation is the relatively small sample size, due to which the results could not be generalized. Moreover, MMT grading is a subjective ordinal scale and may not detect subtle strength differences as precisely as handheld dynamometry. Future studies must be planned by taking these limitations into consideration.

## Conclusions

This prospective study evaluated hip abductor strength in patients undergoing THR, comparing outcomes between the anterolateral and posterior surgical approaches. Muscle power grading improved significantly over a three-month follow-up period on both sides. However, patients who underwent the posterior approach consistently showed greater gains in abductor strength compared to those treated with the anterolateral approach. These findings highlight the potential advantage of the posterior approach in enhancing postoperative abductor function. Nevertheless, the small sample size limits generalizability, and larger, long-term studies are needed to confirm these results.
